# Endoscopic sinus surgery for maxillary sinus mucoceles

**DOI:** 10.1186/1746-160X-2-29

**Published:** 2006-09-06

**Authors:** Fatma Caylakli, Haluk Yavuz, Alper Can Cagici, Levent Naci Ozluoglu

**Affiliations:** 1 Baskent University, Faculty of Medicine, Department of Otorhinolaryngology Head and Neck Surgery, Ankara, Turkey

## Abstract

**Background:**

Maxillary sinus mucoceles are relatively rare among all paranasal sinus mucoceles. With the introduction of endoscopic sinus surgical techniques, rhinologic surgeons prefer transnasal endoscopic management of sinus mucoceles. The aim of this study is to describe the clinical presentation of maxillary sinus mucoceles and to establish the efficacy of endoscopic management of sinus mucoceles.

**Methods:**

Between 2003 and 2005, 14 patients underwent endoscopic sinus surgery for maxillary sinus mucocele. The presenting sign and symptoms, radiological findings, surgical management and need for revision surgery were reviewed.

**Results:**

There were eight males and six females with an age range of 14 to 65. Ten patients complained of nasal obstruction, five of nasal drainage, five of cheek pressure or pain and one of proptosis of the eye and cheek swelling. The maxillary sinus and ipsilateral ethmoid sinus involvement on computed tomographic studies was seen in 4 patients. Four patients had history of endoscopic ethmoidectomy surgery for ethmoid sinusitis and one had Caldwell-Luc operation in the past. Ethmoidectomy with middle meatal antrostomy and marsupialization of the mucocele was performed in all patients. Postoperative follow-up ranged between 8 to 48 months. All patients had a patent middle meatal antrostomy and healthy maxillary sinus mucosa. No patients need revision surgery.

**Conclusion:**

The most common causes of mucoceles are chronic infection, allergic sinonasal disease, trauma and previous surgery. In 64% of the patients of our study cause remains uncertain. Endoscopic sinus surgery is an effective treatment for maxillary sinus mucoceles with a favorable long-term outcome.

## Background

Mucoceles are benign, locally expansile paranasal sinus masses. They are cyst-like structures lined by the mucoperiosteum of the involved sinus [1,2]. Mucoceles are most commonly found in the frontal sinus, with the ethmoid and sphenoid sinuses involved less frequently. Maxillary sinus mucoceles are relatively rare, accounting for 10% or less of all paranasal sinus mucoceles described in the United States or Europe. However, it is more commonly reported in Japan, usually as a long term sequel of Caldwell-Luc surgery [3,4].

Mucoceles are believed to form following obstruction of the sinus ostia, with accumulation of fluid within a mucoperiosteal lined cavity. As mucus continued to be produced within the mucocele, it enlarges gradually, resulting in erosion and remodelling of the surrounding bone [1-6]. Although mucoceles are benign, they can cause significant pathology as a result of their effects on surrounding vital structures, mainly in the periorbital region [7-9]. The most common causes of mucoceles are chronic infection, allergic sinonasal disease, trauma, previous surgery and in some cases cause remains uncertain [1,2].

The treatment of maxillary mucoceles is surgical including external approaches, marsupialization, Caldwell-Luc procedure and endoscopy [1-4,9-11].

In the present study, a series of 14 patients with maxillary sinus mucoceles is reported. The pathogenesis, clinical presentation, endoscopic surgical treatment and differential diagnosis of maxillary mucocele with other cystic expansile masses of the maxilla and need for revision surgery with review of the literature is discussed.

## Methods

This study is a retrospective review of 14 patients with maxillary sinus mucoceles treated at the Department of Otorhinolaryngology in Baskent University Adana Teaching and Research Medical Center between 2003 and 2005. Mucocele was defined in this study as a completely opacified maxillary sinus with evidence of expansion and/or bone erosion. The diagnosis was based on physical examination, including nasal endoscopy, computed tomography (CT) and histopathologic findings. Only patients whose findings on histopathological study of the surgical specimen confirmed the preoperative diagnosis were included in the present study. The medical records were reviewed for patient demographics, presenting symptoms, preoperative CT findings, extent of operation, resolution of symptoms and need for revision surgery.

Follow-up ranged from 8 to 48 months. The surgical outcome was based on the patency of the middle meatal antrostomy, appearance of maxillary sinus mucosa, resolution or persistence of presenting symptoms and need for revision surgery.

## Results

There were 8 males and 6 females ranging from 14 to 65 years. Two patients had bilateral, 6 patients had left and 6 patients had right maxillary sinus mucoceles. On presentation, cheek pressure or pain was reported in 5 patients, nasal drainage in 5, nasal obstruction or congestion in 10. In addition, one patient had proptosis of the eye and cheek swelling. He had no problem with his vision and mobility of the orbit in any direction. Four patients had history of endoscopic ethmoidectomy surgery for ethmoid sinusitis. One patient had Caldwell-Luc operation in the past. None of the patients had history of trauma and environmental allergy. Five patients had history of medical treatment for chronic sinusitis.

**Figure 1:**
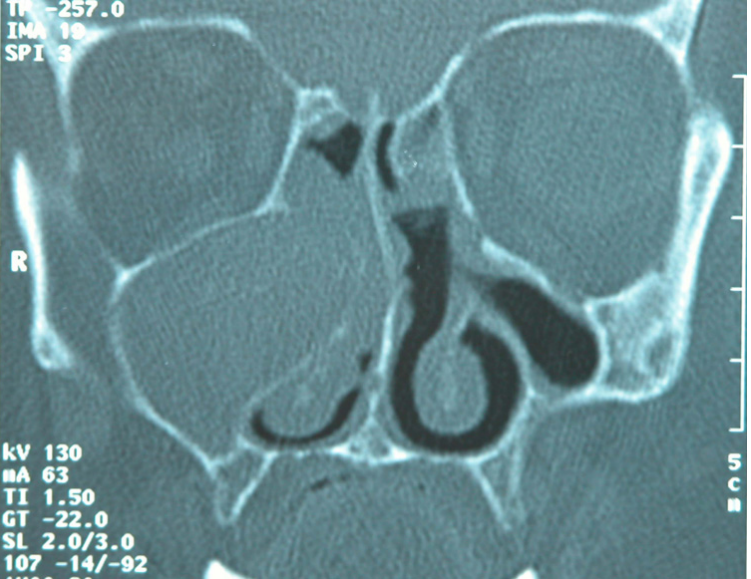
CT scan showing right opacified maxillary sinus with medial bulging causing expansion of the sinus and obstruction of the right nasal cavity.

**Figure 2:**
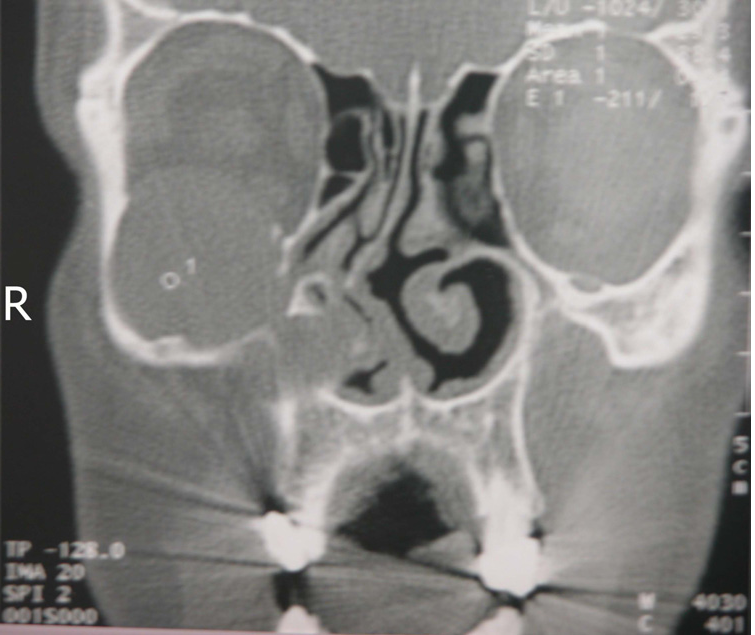
Right maxillary mucocele eroding superior wall of the sinus causing eye proptosis and cheek swelling.

**Figure 3:**
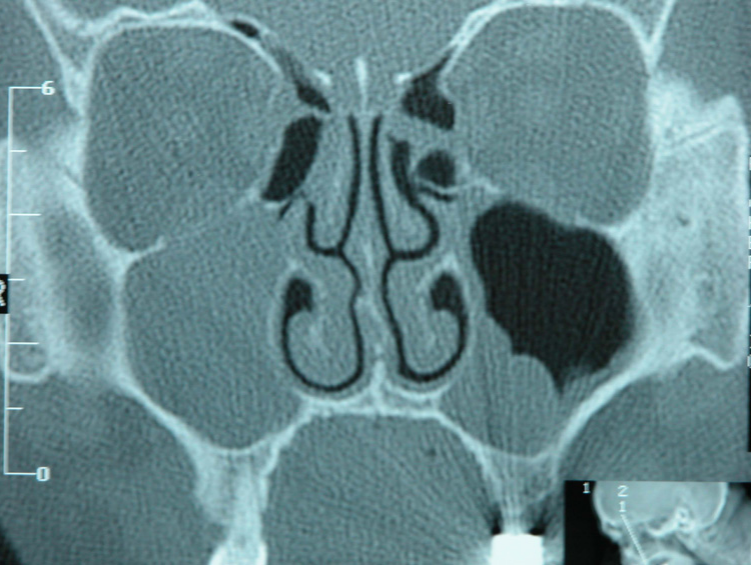
Right maxillary mucocele causing bulging of the uncinate process.

Preoperative CT imaging of the paranasal sinuses was performed in all patients. In all of them, completely opacified maxillary sinuses with homogenous cyst-like lesions were seen and natural ostiums were all obstructed causing the expansion of the sinuses (Fig 1, 2, 3). There was bulging of the medial wall of the maxillary sinus in three patients, eroding the superior wall and bulging into the orbit in one patient. And four patients had mucosal thickening of the ethmoid sinuses.

All the patients underwent endoscopic ethmoidectomy, middle meatal antrostomy and marsupialization with drainage of the mucocele. The contents of the mucocele are evacuated with a curved maxillary sinus suction without the need to totally remove the mucocele lining. Histopathological reports revealed as mucocele lined with pseudostratified columnar epithelium. There were no intraoperative or postoperative complications. Follow-up ranged from 8 to 48 months. All patients reported resolution of their symptoms and no patient required revision surgery. At the last follow-up visit the middle meatal antrostomy was noted to be patent and the maxillary sinus mucosa was observed as normal in all patients (Table 1).

**Table 1:** Patient Characteristics

**Patient No**	**Age**	**Sex**	**Previous Surgery**	**Symptoms**	**Side**	**Surgery**	**Recurrence**	**Follow-up (mo)**
1	56	M	No	Nasal Con	L	ES Eth, MMA	No	12
2	58	M	ES Eth	Nasal Con	L	ES Eth, MMA	No	8
3	47	M	No	Nasal Con Headache	R	ES Eth, MMA	No	11
4	41	F	No	Nasal Con Headache	L	ES Eth, MMA	No	9
5	14	M	No	Nasal Con Cheek Pr	Bilateral	ES Eth, MMA	No	14
6	18	F	No	Nasal Con Headache	L	ES Eth, MMA	No	13
7	46	F	ES Eth	Nasal Con Cheek Pr	Bilateral	ES Eth, MMA	No	10
8	65	F	No	Nasal Dr Cheek Pr	R	ES Eth, MMA	No	9
9	62	M	ES Eth	Nasal Con Nasal Dr	R	ES Eth, MMA	No	10
10	40	M	Cald	Nasal Con, Eye proptosis, Cheek Pr	R	ES Eth, MMA	No	36
11	44	M	No	Nasal Dr Headache	R	ES Eth, MMA	No	24
12	40	F	No	Nasal Dr Headache	L	ES Eth, MMA	No	36
13	51	F	ES Eth	Nasal Dr Cheek Pr	R	ES Eth, MMA	No	48
14	36	M	No	Nasal Con	L	ES Eth, MMA	No	10

Nasal Con: nasal congestion, Nasal Dr: nasal drainage, Cheek Pr: cheek pressure/pain, L: left, R: right, ES Eth: endoscopic ethmoidectomy, MMA: middle meatal antrostomy, Cald: Caldwell

## Discussion

Mucoceles of the paranasal sinuses are benign, cyst-like, expansile lesions lined with a secretory respiratory mucosa of pseudostratified columnar epithelium [1,2]. They are mucoid filled masses and develop after obstruction of the sinus ostium and drainage pattern, which is confirmed by the high incidence of mucoceles in the frontal sinus caused by the variations of the nasofrontal duct [6,9].

Mucoceles grow slowly. Lund and Milroy proposed that the obstruction to sinus outflow in combination with superimposed infection caused the release of cytokines from lymphocytes and monocytes. The cytokine release would stimulate fibroblasts to secrete prostoglandins and collagenases, which in turn could stimulate bone resorption leading to expansion of the mucocele [12].

Maxillary sinus mucoceles are relatively rare accounting for less than 10% of paranasal sinus mucoceles. There are numerous theories about origin and development of maxillary sinus mucoceles, such as chronic infection, allergic sinonasal disease, trauma, previous surgery and in some cases cause remains uncertain. They are more prevalent in Japan, where it is usually reported following Caldwell-Luc maxillary sinusectomy [1,2,9]. Mucoceles that develop following Caldwell-Luc operations are presumed to form as a result of entrapped sinus mucosa. Although one of the theories about development of mucocele is chronic infection, Busaba et al. compared the bacteriology of maxillary sinus mucoceles to chronic sinusitis and reported that the data do not support infection as the main origin of nontraumatic maxillary sinus mucocele [13]. Patients with chronic sinusitis are treated with oral antibiotics preoperatively as in our patient group. During the postoperative period, they are followed up for any symptom and/or need for revision surgery. In our series, 5 patients (36%) had previous surgery (one Caldwell-Luc and 4 endoscopic ethmoid surgery), besides this 9 patients (64%) had no known pathology to cause maxillary mucocele formation.

Mucoceles of the maxillary sinus have been reported previously in the maxillofacial literature [14-17]. The symptoms of mucoceles are related to their expansion and subsequent pressure on and obstruction of surrounding anatomic structures. Antral mucoceles are commonly reported to present as painless bulging of the cheek. Medial expansion of the wall of the maxillary sinus into the nasal cavity displaces the inferior turbinate and causes the nasal obstruction [18]. Superior expansion of the antrum into the inferior orbit can cause displacement of the orbital contents and visual changes. Downward displacement into the area of the alveolus can even cause a loosening of teeth [7-9].

The diagnosis of mucocele is made on the basis of symptoms, imaging and surgical exploration and histological confirmation. The most informative radiologic evaluation is computed tomography. CT scan will show mucocele as a homogenous lesion, which is isodense with brain and no contrast enhancement, unless infected [1,5,19]. There are smooth clear-cut margins of bone erosions occurring in the sinus walls. In contrast, in malignancy the mass is likely to be irregular in shape, with erosion or destruction of the sinus walls, infiltration into the surrounding soft tissues and irregular margins of bone absorption. Magnetic resonance imaging is best reserved for mucocele formation secondary to sinonasal tumors in which lining membrane of the mucocele will enhance after intravenous contrast [5,17]. When the expansion and bone destruction are present the differential diagnosis includes benign and malignant lesions of the paranasal sinuses. Benign lesions include neurofibroma; dermoid, epidermoid, cementifying fibroma; angiofibroma; inverting papilloma and cylindrinoma. Malignant lesions include adenoid cystic carcinoma, plasmocytoma, embryonal rhabdomyosarcoma, lymphoma, schwannoma and tumours of dental origin [5,9]. In the absence of bone erosion, mucoceles must be differentiated from several conditions, including retention cysts, chronic sinusitis, antrachoanal polyp and polyposis of the paranasal cavities [3,5,9].

Retention cysts are common in the maxillary sinus and may be found on imaging studies in approximately 9% of the population. They are thought to form due to obstruction of the ducts of seromucous glands in the sinus lining, which results in an epithelium-lined cyst containing mucous or serous fluid. They develop under mucous membrane of the sinus that explains why they are so thin-walled. Radiographically, the cyst is a rounded, dome-shaped, soft tissue mass, most commonly situated on the flor of the maxillary sinus; it often contains clear, yellowish fluid. Mucoceles are associated with obstruction of the duct or natural ostium of any of the paranasal sinuses and grow under the periosteum. Periosteum contributes to construction of cystic wall, as a result wall of mucocele becomes thick and tough. The growing site of the mucocele is under the periosteum, whereas retention cysts grow under the mucosa of the sinus. This explains that's why retention cysts are non-expanding, well circumscribed, mucosa covered masses, whereas mucoceles exhibit an osteolytic capacity with a tendency to expand along the path of least resistance [3,5,17,20,21].

Antrachoanal polyp is thought to represent hypertrophic maxillary sinus mucosa herniating into the nasal cavity through the natural or accessory ostia. Nasal obstruction is the most common presenting symptom and radiographically appears as an opacity of the involved sinus. They never erode bone [3,9]. Nasal polyps can be single or multiple and may be located in the sinus cavity or the nasal vault. They can cause expansion of the nasal cavity, but do not cause bony erosion [9].

The management of maxillary sinus mucoceles is surgical. Historically, the recommended treatment is complete excision through an open approach that entails Caldwell-Luc sinusectomy, inferior nasoantral window and removal of the mucocele lining. In cases in which significant extension of the mucocele into the facial soft tissues is found, an open approach seems warrented. In cases in which the mucocele is limited to the sinus or extends into the orbit or ethmoid sinus, endoscopic surgery to evacuate the mucocele contents and aerate/drain the mucocele cavity through a wide middle meatal antrostomy is a reliable intervention modality [1,2,10,11].

## Conclusion

There are numerous theories about origin and development of maxillary sinus mucoceles, such as chronic infection, allergic sinonasal disease, trauma and previous surgery. But, as in our series which is 64% of the patients, cause remains uncertain. The diagnosis is usually made by CT imaging of the paranasal sinuses. Endoscopic sinus surgery is an effective treatment modality for maxillary sinus mucocele with favorable long-term outcome.

## Competing interests

The author(s) declare that they have no competing interests.

## Authors' contributions

FC has drafted, prepared the design of the study and the manuscript. HY and CAC carried out the review of the patients' medical records and participated in design of the study. LNO was involved in revising the article for intellectual content details. All authors read and approved the final manuscript.
